# A novel nonslip short-length balloon catheter for endoscopic papillary large balloon dilation

**DOI:** 10.1055/a-2499-7370

**Published:** 2025-01-14

**Authors:** Tadahisa Inoue, Rena Kitano, Tomoya Kitada, Kazumasa Sakamoto, Satoshi Kimoto, Jun Arai, Kiyoaki Ito

**Affiliations:** 1 12703 Department of Gastroenterology, Aichi Medical University, Nagakute, Japan

Endoscopic papillary large balloon dilation (EPLBD) is an effective technique for removing large or stacked bile duct stones 1 . However, slippage during dilation is a common challenge, often prolonging procedures, necessitating multiple attempts, and increasing the risk of adverse events. This issue is more pronounced with larger EPLBD balloons compared to standard endoscopic papillary balloon dilation. Although longer balloons may reduce slippage, they can cause unnecessary dilation beyond the papilla and are challenging to use in cases where stones are located near the papilla. Additionally, maintaining an adequate distance between the endoscope and the papilla can be challenging during the procedure.

To address these limitations, we developed a novel EPLBD balloon catheter (RIGEL; Japan Lifeline, Tokyo, Japan) ( Fig. 1 ), with a length of 25 mm—shorter than conventional EPLBD balloons, which are typically 40–50 mm. This catheter includes an elastic band at its middle, which delays the expansion of the central segment, effectively preventing slippage 2 . Its tapered ends reduce the risk of stone entrapment between the balloon and the bile duct wall, particularly in cases involving impacted stones in the distal bile duct.

**Fig. 1:**
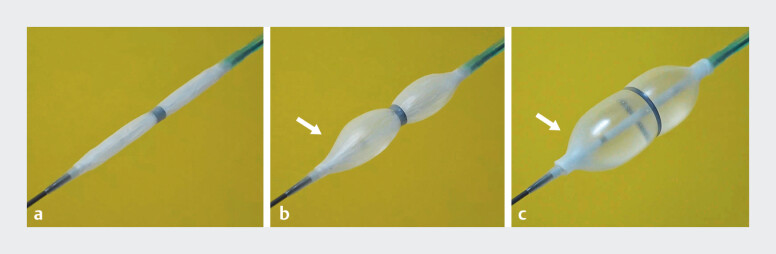
A novel endoscopic papillary large balloon dilation catheter (RIGEL; Japan Lifeline, Tokyo, Japan) with a length of 25 mm, which is shorter than the conventional balloons that typically have a length of 40–50 mm. **a–b** The balloon features an elastic band in the middle ( **a** ), causing the central part to expand with a delay to prevent slippage ( **b** ). **c** Its tapered ends (arrow) reduce the risk of stone entrapment between the balloon and bile duct wall, particularly in cases of impacted stones in the distal bile duct.

A 75-year-old man presented with jaundice and cholangitis caused by common bile duct stones. After successful bile duct cannulation, cholangiography revealed a large stone impacted in the distal end of the common bile duct. Following endoscopic sphincterotomy, a RIGEL balloon (12×25 mm) was inserted, with the band positioned at the papilla. The balloon was inflated to 6 atm, and its central segment expanded with a delay, allowing precise papillary dilation on the first attempt without slippage. Although the stone was located near the papilla, it shifted toward the liver during dilation without becoming trapped ( Fig. 2 , Video 1 ). The stone was subsequently removed using a basket and balloon catheters. The patient’s symptoms resolved rapidly, and no adverse events were observed.

**Fig. 2:**
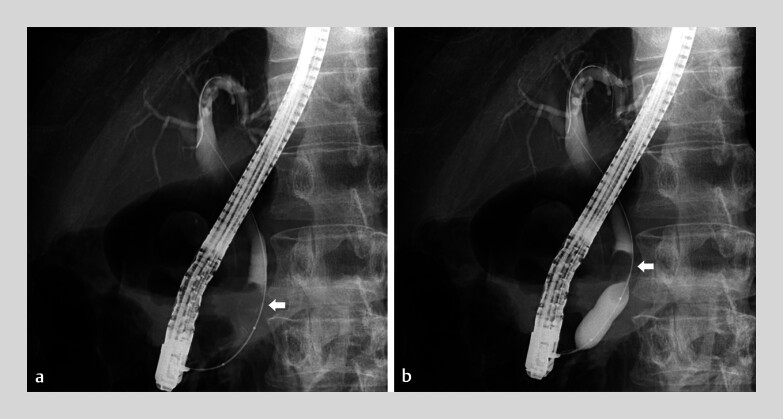
**a** After successful bile duct cannulation, cholangiography revealed a large stone (arrow) impacted in the distal end of the common bile duct. Following endoscopic sphincterotomy, the RIGEL balloon (12×25 mm) was inserted, and the band was positioned at the papilla. The balloon was subsequently inflated to 6 atm. The central part of the balloon expanded with a delay to ensure appropriate papillary dilation on the first attempt without slippage. **b** Although the stone was located near the papilla, it migrated toward the liver (arrow) during the dilation without becoming trapped.

Endoscopic papillary large balloon dilation using a novel nonslip short-length balloon in a 75-year-old patient with a large impacted stone in the distal common bile duct.Video 1

Endoscopy_UCTN_Code_TTT_1AR_2AC

## Notes

Endoscopy E-Videos https://eref.thieme.de/e-videos
*E-Videos* is an open access online section of the journal *Endoscopy* , reporting on interesting cases and new techniques in gastroenterological endoscopy. All papers include a high-quality video and are published with a Creative Commons CC-BY license. Endoscopy E-Videos qualify for HINARI discounts and waivers and eligibility is automatically checked during the submission process. We grant 100% waivers to articles whose corresponding authors are based in Group A countries and 50% waivers to those who are based in Group B countries as classified by Research4Life (see: https://www.research4life.org/access/eligibility/ ). This section has its own submission website at https://mc.manuscriptcentral.com/e-videos .

## References

[1] Facciorusso A Gkolfakis P Ramai D Endoscopic treatment of large bile duct stones: a systematic review and network meta-analysis Clin Gastroenterol Hepatol 2023 21 33 4.4E10 34666153 10.1016/j.cgh.2021.10.013

[2] Inoue T Kutsumi H Ibusuki M A novel non-slip banded balloon catheter for endoscopic sphincteroplasty: an ex vivo and in vivo pilot study Sci Rep 2023 13 4032 10.1038/s41598-023-31206-6 36899107 PMC10006090

